# A Rare Case of Brain Metastasis of Gastric Neuroendocrine Carcinoma

**DOI:** 10.7759/cureus.83417

**Published:** 2025-05-03

**Authors:** Ryota Hori, Naoki Shinojima, Kenji Fujimoto, Daiki Yoshii, Hiroyuki Uetani, Yoshiyuki Fukugawa, Fumi Kawakami, Yoshiki Mikami, Natsuo Oya, Toshinori Hirai, Akitake Mukasa

**Affiliations:** 1 Department of Neurosurgery, Faculty of Life Sciences, Kumamoto University, Kumamoto, JPN; 2 Department of Neurosurgery, National Hospital Organization Kumamoto Medical Center, Kumamoto, JPN; 3 Department of Diagnostic Pathology, Kumamoto University Hospital, Kumamoto, JPN; 4 Department of Diagnostic Radiology, Faculty of Life Sciences, Kumamoto University, Kumamoto, JPN; 5 Department of Radiation Oncology, Faculty of Life Sciences, Kumamoto University, Kumamoto, JPN; 6 Department of Pathology and Cell Biology, University of the Ryukyus, Okinawa, JPN

**Keywords:** gastric cancers, gastric neuroendocrine carcinoma, neuroendocrine tumors, small cell neuroendocrine carcinoma, solitary brain metastasis

## Abstract

Gastric neuroendocrine carcinoma (small cell neuroendocrine carcinoma [small cell NEC]) is a rare type of gastric cancer that is rarely encountered in clinical practice. Although solitary brain metastasis from gastric cancer is exceptionally rare, we present a case of solitary brain metastasis of gastric NEC.

The patient was a 71-year-old man who had undergone surgery and chemotherapy for gastric NEC two years prior. During follow-up, computed tomography (CT) scans of the neck, chest, abdomen, and pelvis, along with tumor marker evaluations, revealed no evidence of metastasis. However, he developed left homonymous hemianopsia, prompting a head CT and magnetic resonance imaging (MRI). Imaging revealed a subcortical hemorrhage in the right temporal lobe and tumors in two locations: the right temporal lobe and the upper surface of the cerebellum. Tumor resection and hematoma removal were performed for diagnostic and decompressive purposes. Histopathological examination confirmed the diagnosis of brain metastasis from gastric NEC.

Brain metastases from gastric cancer are rare, and to the best of our knowledge, only one English-language case report of solitary brain metastasis from gastric NEC has been published. We report this case and provide a discussion based on the available literature.

## Introduction

Gastric neuroendocrine carcinoma (small cell neuroendocrine carcinoma [small cell NEC]) is a rare histological subtype of gastric cancer; however, its incidence has been increasing in recent years [1]. Gastroenteropancreatic neuroendocrine neoplasms are broadly categorized into well-differentiated neuroendocrine tumors and poorly differentiated neuroendocrine carcinomas (NECs), the latter being associated with markedly aggressive behavior and poor clinical outcomes [2,3]. Brain metastasis from gastric adenocarcinoma is uncommon, and standard follow-up protocols typically exclude routine brain imaging. Here, we report a rare clinical case of solitary brain metastasis that occurred approximately two years after initial curative treatment for gastric NEC, consisting of surgical resection and adjuvant chemotherapy. We present this case along with a review of the relevant literature, highlighting the need for heightened clinical vigilance regarding neurological complications in patients with previously treated gastric NEC.

## Case presentation

A 71-year-old man presented with left homonymous hemianopsia, without other focal neurological deficits. Two years earlier, he had undergone total gastrectomy, splenectomy, and pancreatectomy for gastric NEC, followed by four courses of CE (CBDCA/VP-16) therapy. A cervical-thoracic-abdominopelvic CT scan performed immediately prior to the onset of visual field disturbance revealed no evidence of recurrence. Tumor markers were also within normal limits at that time - neuron-specific enolase (NSE): 9.1 ng/mL (reference <10 ng/mL) and pro-gastrin-releasing peptide (ProGRP): 17.1 pg/mL (reference <81 pg/mL) - and remained within normal ranges throughout the follow-up period.

**Figure 1:**
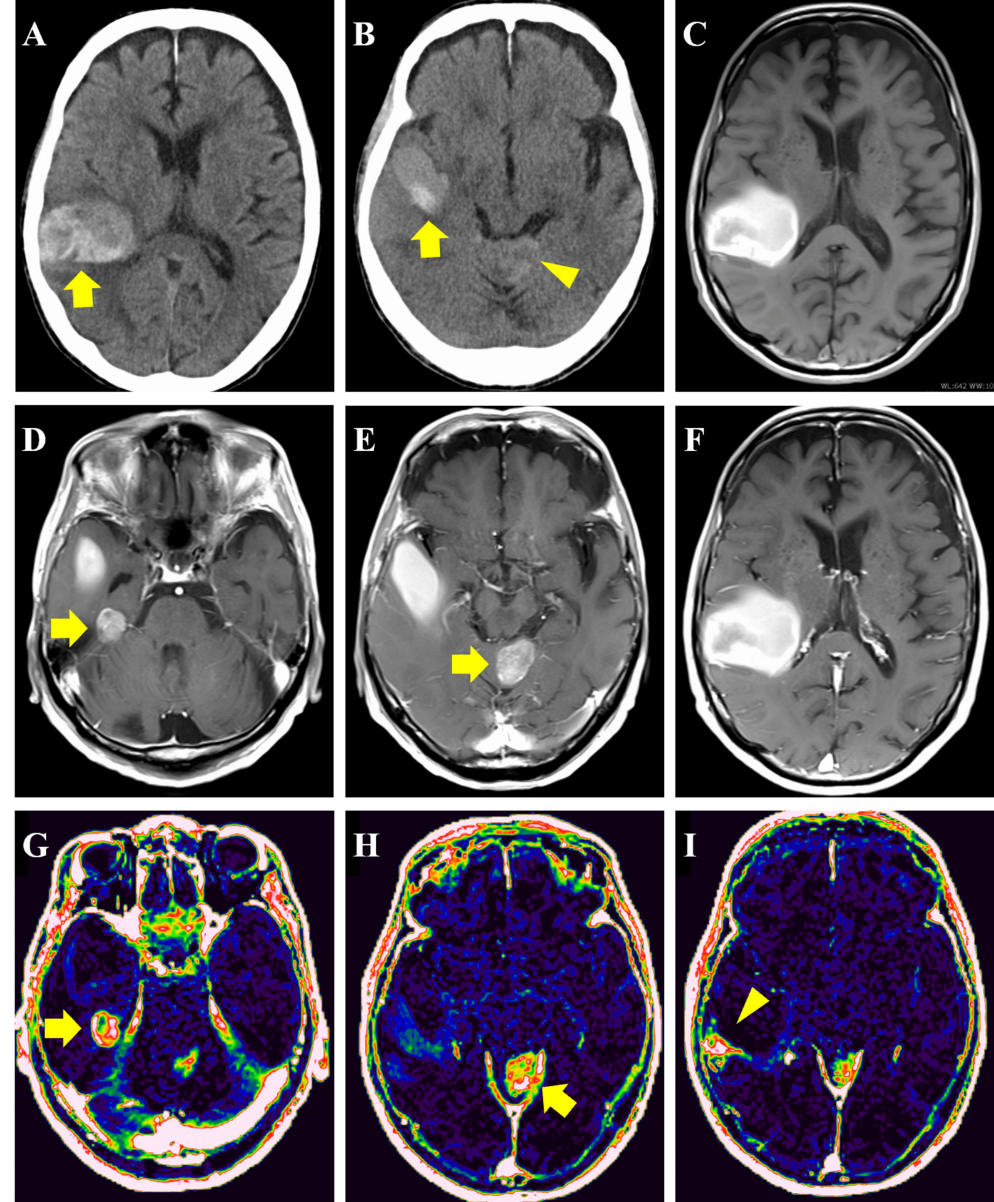
Radiological findings at disease onset A, B: Head CT showing subcortical hemorrhage (arrows) and a faint high-density area suspicious for tumor (arrowhead). C: T1-weighted MRI; D-F: Gadolinium-enhanced T1WI. D, E: Well-defined enhancing lesions (arrows). F: No enhancement. G-I: DCE-MRI–derived Ve maps. G, H: Elevated perfusion in enhancing lesions (arrows). I: Additional perfusion elevation (arrowhead) without enhancement, suggestive of another neoplastic lesion. CT, computed tomography; T1WI, T1-weighted image; DCE-MRI, dynamic contrast-enhanced magnetic resonance imaging; Ve, extravascular extracellular space volume per unit tissue volume.

Following the onset of visual field disturbance, head CT and MRI revealed a subcortical hemorrhage in the right temporal lobe (Figure 1A, 1B, 1C). Contrast-enhanced MRI showed a hemorrhagic tumor, 23 mm in diameter, on the medial dorsal surface of the right temporal lobe (Figure 1D), 27 mm in diameter, on the superior surface of the cerebellar vermis (Figure 1E), but no contrast enhancement in the right temporal-parietal lobes (Figure 1F). Extravascular extracellular space volume per unit tissue volume (Ve) imaging derived from dynamic contrast-enhanced MRI revealed elevated perfusion in the regions corresponding to the enhanced mass lesions (Figure 1G, 1H). An additional area of elevated perfusion was observed dorsal to the hemorrhagic lesion in the right temporal-parietal lobes (Figure 1I), with no contrast enhancement, suggesting a possible neoplastic lesion. In the whole-body fluorodeoxyglucose positron emission tomography (FDG-PET), high accumulation was observed inside the right temporal lobe hematoma. On the other hand, the tumor in the cerebellar vermis showed the same level of accumulation as the surrounding tissue, and no other abnormal accumulation was detected in the trunk (data not shown).

**Figure 2:**
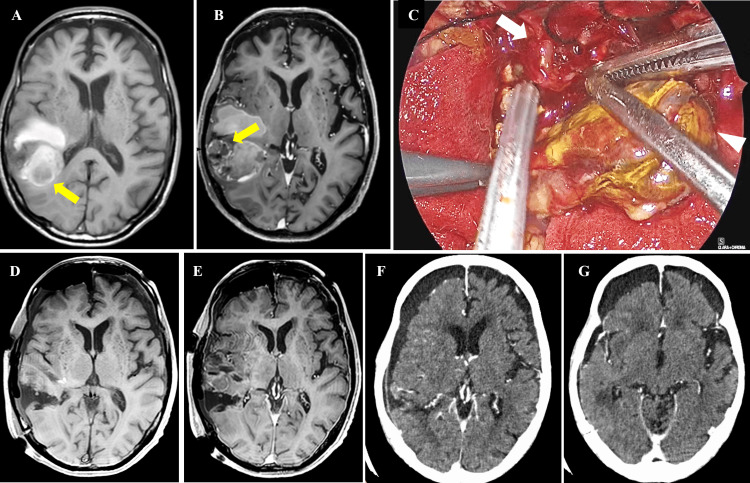
Radiological images during the disease course and a representative intraoperative photograph A, B: Shows MRI taken approximately 2.5 months after the onset of brain metastasis. A: New hemorrhage in the right temporal lobe on T1WI (arrow). B: A new enhancement corresponding to the previously noted elevated perfusion area (arrow), confirming the presence of a tumor. C: Shows neuroendoscopic intraoperative findings. Arrow: tumor adjacent to the hematoma, arrowhead: hematoma component. D, E: Shows postoperative MRI. D: T1WI. E: Gd-T1WI. F, G: Shows contrast-enhanced CT images obtained 75 days after the pathological diagnosis of brain metastasis. MRI, magnetic resonance imaging; T1WI, T1-weighted image; CT, computed tomography.

The patient initially opted for follow-up imaging through a shared decision-making process as part of advance care planning (ACP). An MRI performed 2.5 months later revealed rebleeding in the right temporal lobe (Figure 2A) and a new enhancement corresponding to the previously noted elevated perfusion area (Figure 2B), confirming the presence of a tumor. Following further discussion within the ACP framework, the patient elected to undergo a minimally invasive surgical procedure using a neuroendoscope for diagnostic and decompressive purposes, with planning subsequent radiotherapy for local control (Figure 2C). A small craniotomy was performed to remove the hematoma and the tumor adjacent to the hematoma (Figure 2D, 2E).

Histopathological findings

**Figure 3:**
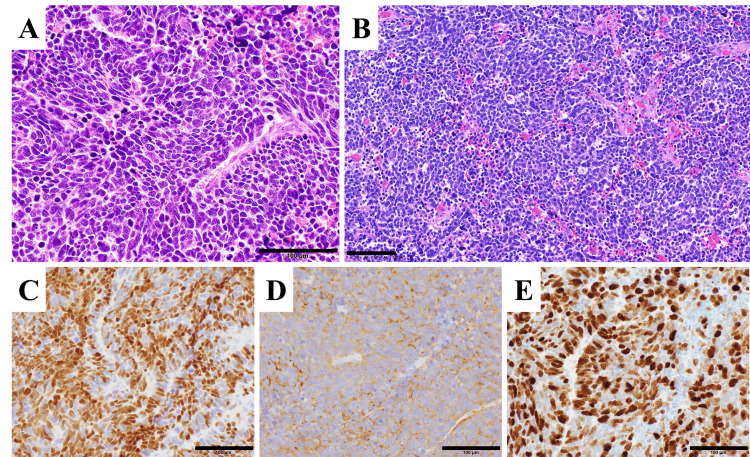
Histopathological findings A, B: Shows H&E staining. A: Brain tumor tissues. B: Specimens of primary small cell neuroendocrine carcinoma of the stomach. C-E: Shows immunohistochemical findings of brain tumor tissues. C: INSM1. D: Synaptophysin. E: Ki-67. Each scale bar = 100 μm. INSM1, Insulinoma-associated protein 1.

In the resected brain tissue, the tumor cells demonstrated a high nucleus-to-cytoplasm ratio and hyperchromatic nuclei arranged in a solid sheet-like growth pattern showing nuclear molding (Figure 3A). Numerous mitotic figures were observed. The histology was identical to that of the previously resected gastric small cell NEC (Figure 3B). Immunohistochemically, tumor cells of the resected brain tissue were positive for neuroendocrine markers: INSM1 (insulinoma-associated protein 1) and synaptophysin (Figure 3C, 3D). The Ki-67 proliferation index was elevated, with an average of 72.1% (Figure 3E). Based on these findings, the brain tumor was diagnosed as a metastatic lesion originating from the primary gastric small cell NEC.

Postoperative course and outcome

Postoperatively, the patient underwent whole-brain irradiation including the cerebellar lesion (30 Gy in 10 fractions), followed by local boost irradiation to each lesion (15 Gy in five fractions). Follow-up whole-body CT confirmed no metastases in other regions. However, complications such as aspiration pneumonia gradually worsened his general condition, leading to bedridden status, and he was transferred to a local hospital for palliative care. Despite the absence of new lesions and no progression of the unresected cerebellar vermis lesion on contrast-enhanced head CT images obtained 75 days after the pathological diagnosis of brain metastasis (Figure 2F, 2G), the patient contracted COVID-19 and died 110 days after the diagnosis. As shown in Table 1, summarizing the entire clinical course, the patient died 185 days after the onset of brain metastasis, and the cause of death was COVID-19, not tumor progression.

**Table 1:** Timeline of clinical course Timeline of the patient’s clinical course, including key symptoms, imaging and pathological findings, and interventions. Imaging figures are referenced in parentheses. NEC, neuroendocrine carcinoma; CT, computed tomography; NSE, neuron-specific enolase; ProGRP, pro-gastrin-releasing peptide; MRI, magnetic resonance imaging; FDG-PET, fluorodeoxyglucose positron emission tomography.

Timepoint	Key Symptoms/Clinical Events	Imaging/Laboratory Findings (Figure Reference)	Treatment/Management
2 years prior	Diagnosed with gastric NEC	Histopathology: gastric small cell NEC	Total gastrectomy, splenectomy, pancreatectomy + adjuvant chemotherapy (CBDCA/VP-16, 4 cycles)
Follow-up period	Asymptomatic	Cervico-thoraco-abdominopelvic CT: no recurrence; tumor markers (NSE and ProGRP): within normal range	Regular follow-up
Day 0	Onset of left homonymous hemianopsia	Head CT: subcortical hemorrhage in right temporal lobe (Figure 1A, 1B). MRI: tumors in the right temporal lobe and cerebellar vermis (Figure 1D, 1E). FDG-PET: no abnormal accumulation in the trunk	Follow-up under advance care planning
Day 70	Rebleeding confirmed	MRI: new enhancement at high-perfusion area, confirming tumor (Figures 1I, 2B)	—
Day 75	Diagnosed with brain metastasis from gastric NEC	Histopathology: brain metastasis from gastric NEC, Ki-67 index = 72.1% (Figure 3)	Neuroendoscopic resection of hematoma and tumor in the right temporal lobe
Postoperative period	No neurological deterioration	—	Whole-brain irradiation (30 Gy/10 fx) + local boost (15 Gy/5 fx)
	Gradual systemic deterioration with pneumonia	Whole-body CT: evidence of pneumonia	Treatment for pneumonia
Day 120	Transfer to another hospital	—	Transition to palliative care
Day 150	No disease progression	Head CT: no recurrence in the right temporal lobe, disappearance of the unresected enhancing lesion in the cerebellar vermis (Figure 2F, 2G), and no new lesions	Continuation of palliative care
Day 170	COVID-19	Whole-body CT: worsening pneumonia	Treatment for COVID-19-related pneumonia
Day 185	Death due to COVID-19	—	—

## Discussion

Solitary brain metastasis of gastric NEC is extremely rare. We searched PubMed for reports of solitary brain metastasis from gastric NEC reported up to 2024 using the following keywords: “small cell, carcinoma, gastric, stomach, metastatic brain tumor, neuroendocrine”. We identified only one previously reported case, described by Bugalho et al. [4]. A notable difference between our case and that of Bugalho et al. lies in the clinical trigger that led to the discovery of brain metastasis. In our patient, the brain lesion was identified only after the development of neurological symptoms following total gastrectomy for a previously diagnosed gastric NEC. In contrast, in Bugalho’s report, neurological symptoms were the initial presentation, prompting imaging that subsequently revealed both the brain metastases and the gastric primary tumor. This contrast underscores the variability in clinical presentation and timing of brain metastasis in gastric NEC and highlights the importance of maintaining neurological vigilance even after apparent curative treatment of the primary tumor.

In a study by Akimoto et al., brain metastases were found in 31 out of 302 neuroendocrine tumor cases (10.3%), including NEC. However, more than 80% of the primary tumors originated in the lung, with no cases of gastric origin [5]. Gastric cancer rarely presents with solitary brain metastases, mainly because, by the time brain metastases are detected, multi-organ metastases have typically already progressed [6].

Gastric NEC is a rare subtype of gastric cancer, accounting for approximately 0.1-0.6% of all cases, but due to advances in diagnostic technology and the establishment of a disease concept, its incidence has been increasing in recent years [1]. As a result, it is possible that data on cases of brain metastasis of gastric NEC will be accumulated in the future.

Because brain metastasis is rare in gastric cancer, this case involved routine follow-up with regular NSE and ProGRP tumor marker measurements and whole-body CT scans, excluding the brain. Neither the tumor markers nor the CT scans suggested recurrence, and the brain metastasis was diagnosed following a cerebral hemorrhage. Unlike conventional gastric cancer, gastric NEC is associated with genomic abnormalities such as TP53 and RB1 and exhibits histological similarities to small cell lung carcinoma (SCLC) [7]. Gastric small cell NEC is also pathologically and biologically similar to SCLC [4]. As SCLC is known to have a high propensity for brain metastases, gastric NEC may also have a higher frequency of brain metastases compared to conventional gastric cancer. Furthermore, monitoring tumor markers such as NSE and ProGRP is considered useful for managing pancreatic and gastrointestinal NEC [8,9]. However, in cases like this, where the primary lesion is well-controlled and metastasis is limited to the brain, tumor markers may fail to detect brain metastases. This underscores the need for alternative methods to detect brain metastases beyond tumor markers.

Regarding prognosis, the median survival time for gastric cancer patients diagnosed with brain metastases is typically only a few months, indicating a poor prognosis [10]. However, early detection of brain metastases and effective local control can enable the continuation of chemotherapy and may extend survival [10]. In this case, tumor markers did not contribute to the early detection of brain metastases, emphasizing the importance of head CT as a complementary diagnostic tool. Thus, head CT and MRI are considered useful for the early detection of brain metastases during the follow-up of gastric NEC and may contribute to prolonged survival.

## Conclusions

Solitary brain metastasis from gastric NEC is extremely rare, with only one previously reported English-language case, although other non-solitary cases may exist beyond the scope of our literature search. Given its biological similarity to SCLC, gastric NEC may have a higher propensity for brain metastasis compared to conventional gastric cancer. Incorporating head CT or MRI into follow-up protocols may be beneficial in patients with gastric NEC who develop new neurological symptoms. Further accumulation of cases and systematic data analysis will be essential to better characterize the metastatic behavior and optimize surveillance strategies for this aggressive tumor type.
